# Supplementary material for: Liquefied Petroleum Gas or Biomass for Cooking and Effects on Blood Pressure: Results from the Household Air Pollution Intervention Network (HAPIN) Trial
Source: medRxiv. 2025 Aug 19:2025.08.15.25333780. Preprint. [Version 1] doi: 10.1101/2025.08.15.25333780 (PMC12393639; doi:10.1101/2025.08.15.25333780)
Supplement: Supplement 1 [file media-1.zip › HAPIN BP supplemental tables S1_S2.pdf]

Table S1. Measured effect of the HAPIN intervention on blood pressure using primary and secondary analytical models across four participant subsets with varying levels of medication use. All models evaluate differences in blood pressure between study arms. Model 1 utilizes mean blood pressure after randomization as the outcome and adjusts for centered mean baseline blood pressure and randomization strata. Model 2 uses the change in blood pressure from baseline to the final post-randomization visit as the outcome and adjusts for randomization strata but not baseline blood pressure. Model 3 utilizes a repeated measures analysis incorporating a random effect for women to compare average post-randomization blood pressure between study arms. Model 4 compares the time trend (slope) of blood pressure changes between study arms using a repeated measures analysis with a random intercept for women, coding consecutive visits numerically from baseline onward. All models were applied to three participant subsets: The "after" subset excluded measurements at and after first report of medication usage; the "BL" subset excluded all participants reporting medication use at baseline only; and the "Any" subset excluded participants who reported medication use at any timepoint. The model and subsets used in the primary analysis are indicated by an asterisk.

		Model 1* Post-Randomization Mean Difference			Model 2 Change (Baseline to 18-Months Post-Randomization)			Model 3 Repeated measures + Random Effects			Model 4 Repeated measures + Slopes Comparison		
	Exclusion	n	Est.	95% CI	n	Est.	95% CI	n	Est.	95% CI	n	Est.	95% CI
SBP	After	352	0.08	(-1.68, 1.84)	NA	NA	NA	1262	0.83	(-1.86, 3.52)	1621	0.02	(-0.49, 0.53)
	BL	359	0.23	(-1.52, 1.98)	248	-0.11	(-3.46, 3.24)	1304	0.82	(-2.03, 3.66)	1663	0.07	(-0.45, 0.6)
	Any*	342	-0.56	(-2.19, 1.07)	248	0.20	(-3.03, 3.42)	1244	-0.01	(-2.52, 2.5)	1586	-0.01	(-0.52, 0.49)
DBP	After	352	-0.85	(-2.1, 0.4)	NA	NA	NA	1262	0.13	(-1.54, 1.8)	1621	-0.30	(-0.67, 0.08)
	BL	359	-0.91	(-2.15, 0.33)	248	-1.82	(-4.38, 0.75)	1304	0.09	(-1.64, 1.82)	1663	-0.23	(-0.6, 0.14)
	Any*	342	-1.21	(-2.41, -0.01)	248	-1.90	(-4.52, 0.71)	1244	-0.14	(-1.78, 1.49)	1586	-0.32	(-0.69, 0.06)
PP	After	352	1.04	(-0.14, 2.22)	NA	NA	NA	1262	0.68	(-0.99, 2.34)	1621	0.33	(-0.05, 0.7)
	BL	359	1.20	(0.03, 2.37)	248	1.71	(-0.98, 4.4)	1304	0.74	(-1, 2.47)	1663	0.31	(-0.08, 0.71)
	Any*	342	0.67	(-0.45, 1.79)	248	2.10	(-0.49, 4.68)	1244	0.16	(-1.4, 1.71)	1586	0.32	(-0.05, 0.69)
MAP	After	352	-0.74	(-2, 0.52)	NA	NA	NA	1262	0.35	(-1.56, 2.26)	1621	-0.20	(-0.58, 0.19)
	BL	359	-0.56	(-1.88, 0.76)	248	-1.25	(-3.8, 1.31)	1304	0.31	(-1.7, 2.32)	1663	-0.14	(-0.52, 0.25)
	Any*	342	-1.02	(-2.27, 0.23)	248	-1.20	(-3.76, 1.35)	1244	-0.12	(-1.95, 1.72)	1586	-0.22	(-0.6, 0.16)

Table S2: Measured effect of the HAPIN intervention on blood pressure over time within control and intervention arms. Estimates represent the change in mean blood pressure over time (slope) within each study arm, using a repeated measures analysis with a random intercept for women, with visits coded sequentially from baseline onward (i.e., Model 4 from Table S1, stratified by study arm). All models were applied to the three participant subsets defined in Table S1.

		Model 4 (Control)			Model 4 (Intervention)		
	Exclusion	n	Est.	95% CI	n	Est.	95% CI
SBP	After	795	-0.48	(-0.84, -0.12)	826	-0.47	(-0.83, -0.11)
SBP	BL	815	-0.56	(-0.94, -0.19)	848	-0.49	(-0.86, -0.12)
SBP	Any	783	-0.40	(-0.76, -0.04)	803	-0.41	(-0.77, -0.05)
DBP	After	795	0.03	(-0.23, 0.29)	826	-0.27	(-0.53, -0.01)
DBP	BL	815	0.01	(-0.25, 0.28)	848	-0.22	(-0.48, 0.04)
DBP	Any	783	0.07	(-0.2, 0.33)	803	-0.25	(-0.51, 0.01)
pulse	After	795	-0.53	(-0.8, -0.27)	826	-0.21	(-0.47, 0.06)
pulse	BL	815	-0.58	(-0.85, -0.3)	848	-0.26	(-0.54, 0.01)
pulse	Any	783	-0.46	(-0.73, -0.2)	803	-0.14	(-0.41, 0.12)
MAP	After	795	-0.14	(-0.41, 0.13)	826	-0.34	(-0.61, -0.07)
MAP	BL	815	-0.18	(-0.45, 0.1)	848	-0.31	(-0.59, -0.04)
MAP	Any	783	-0.09	(-0.36, 0.18)	803	-0.31	(-0.58, -0.04)
